# Stress Cardiomyopathy Triggered by Status Epilepticus Secondary to Herpes Simplex Virus Encephalitis: Case Report and Literature Review

**DOI:** 10.7759/cureus.20615

**Published:** 2021-12-22

**Authors:** Rafail Beshai, Jeffrey J Lee

**Affiliations:** 1 Internal Medicine, Jefferson Health, Stratford, USA; 2 Cardiology, Cooper University Hospital, Camden, USA

**Keywords:** takotsubo, herpes simplex virus type 1, epileptic seizures, hsv-1, stress-related cardiomyopathy

## Abstract

Stress cardiomyopathy (SCM) occurs in approximately 1% of patients presenting with troponin-positive suspected acute coronary syndrome (ACS). We present here a 50-year-old female who presented to the emergency department (ED) with altered mental status. In the hospital, she was found to have status epilepticus (SE) secondary to Herpes simplex virus encephalitis. Her hospital stay was complicated by high troponins and a transthoracic echocardiogram showed reduced ejection fraction and wall motion abnormality. Repeat echo five days later showed normal ventricular systolic function with no wall motion abnormality. Extensive ischemia workup was negative. A diagnosis of stress cardiomyopathy has been made. We urge physicians to include SCM in their differential diagnosis especially in cases of status epilepticus in order to avoid invasive procedures and for better management of patients.

## Introduction

Herpes simplex virus 1 (HSV-1) encephalitis is the most common cause of fatal sporadic encephalitis in the United States [1]. Status epilepticus (SE) secondary to HSV encephalitis represents a relatively common neurologic emergency with significant associated morbidity and mortality [2]. Stress cardiomyopathy (SCM) is an unusual form of acute left ventricular dysfunction that usually recovers spontaneously within days or weeks [3]. The combined involvement of SCM, SE, and HSV encephalitis is very rare. Clinicians should be aware of this unique concurrence to aid with proper diagnosis and management.

## Case presentation

A 50-year-old female, with a history of paroxysmal atrial flutter on propafenone and Eliquis, seizure disorder, and hypertension, presented to the emergency department with altered mental status. She presented to the hospital after her family heard a thump and found her unconscious. She regained consciousness after about seven to eight minutes but was confused. According to her family members, the patient was having fever and headaches for the past day. In the ED, the patient was found to have a fever with a temperature of 101.2F, tachycardia, leukocytosis of 12.5, and lactic acid of 1.7. The physical exam was significant for severe fluent aphasia, multiple paraphasic errors with difficulties with naming. Neurology did not feel that she was a candidate for thrombolysis, as she was admitted for questionable stroke or postictal state while being on Eliquis. CT scan of her head showed no acute intracranial hemorrhage, transcortical infarction, mass effect, or midline shift. Computed tomography angiography (CTA) was negative for any occlusion. MRI of the brain was negative for any stroke but showed left medial temporal hyperintensity. The patient was also put on continuous electroencephalogram (cEEG), which noted as many as 10 seizures per hour lasting from 10-20 seconds. She was loaded with midazolam bolus and was initiated on 0.1 mg/kg/hr for seizure suppression. She was also given 1500 mg q12 of levetiracetam in addition to her home lamotrigine 150 mg in the morning and 225 mg at night.

The patient was upgraded to the ICU for intubation, as she was not protecting her airway and for close monitoring of her status epilepticus activity. Even though she was on three drugs for seizure, cEEG showed one more seizure that lasted 40 seconds. The patient was first placed on empiric antibiotic treatment with Bactrim, acyclovir, vancomycin, and ceftriaxone. A lumbar puncture was performed. The cerebrospinal fluid polymerase chain reaction (PCR) panel detected Herpes simplex virus 1 with predominant lymphocytosis. Cerebrospinal fluid (CSF) analysis showed RBC: 4, glucose: 64, protein: 58, and lymphocytes: 99. All antibiotics were stopped and only acyclovir was continued. Given that she was seizure-free after Day 6 of her admissions, midazolam was discontinued. The patient was successfully extubated, was stable for a downgrade to step down, and was eventually discharged.

Her hospital stay was complicated by increased high-sensitivity troponins that trended up from 155 to 768 (normal value <14) on the initial workup in the ED on the day of arrival. Cardiology was consulted. A transthoracic echo on the same day showed left ventricular function to appear segmentally reduced with mid-anteroseptal and inferior and inferoseptal hypokinesis (Video 1). EKG showed normal sinus rhythm with no ST-segment changes. The patient did not have any history of coronary artery disease in the past with a negative family history of ischemic heart diseases. The patient was started on guideline-directed medical therapy for non-ST elevation myocardial infarction (NSTEMI) and was placed on a high-dose heparin drip and beta-blocker. Propafenone was discontinued given her troponin spill and the possibility of coronary disease. Amiodarone was started. Cardiology decided to repeat a contrast transthoracic echo five days later to better assess the left ventricular wall motion. It showed normal left ventricular systolic function without segmental wall motion abnormalities with an estimated ejection fraction of 60-65% (Video 2). A coronary angiogram was not performed due to the risk-benefit analysis of continued resistant seizure activity on triple antiepileptic therapy. The combination of our knowledge that seizures can cause SCM, quick resolution of her echocardiogram findings, and conversion to benzodiazepine coma to stop all seizure activity precluded the ability to perform a coronary angiogram. However, an Extensive ischemia workup was done including a Persantine thallium perfusion scan that showed normal left ventricular perfusion that is negative for ischemia without segmental wall motion abnormality. This test was done after complete recovery from status epilepticus.

**Video 1:** Initial transthoracic echo Apical two-chamber view showing left ventricular function to be segmentally reduced with mid-anteroseptal and inferior and inferoseptal hypokinesis

**Video 2:** Second transthoracic echo done five days later Apical two-chamber view of transthoracic echo showing no wall motion abnormality

## Discussion

Stress cardiomyopathy is a relatively rare disease with an incidence rate of 2.3-3 in 100,000 [4]. The relationship between SCM and status epilepticus is not understood in detail. A recent study showed that the frequency of SCM in seizure-related hospitalizations among adults does not exceed 0.1% [5]. However, there is a lack of data for the status epileptic state leading to SCM. Some specific factors could explain the difficulties in recognizing SCM in patients with SE: inability to complain of chest discomfort in the presence of altered mental state and creatine phosphokinase elevation as a result of convulsive seizures.

Although some triggers, including emotional stress and status epilepticus, are identified, SCM remains a medical mystery. It is unclear as to why status epilepticus leads to cardiac dysfunction in some patients but not others. Although the precise etiology of stress cardiomyopathy is not known, the most plausible cause responsible is the sudden release of stress hormones, such as norepinephrine, epinephrine, and dopamine, causing cardiac stunning [6]. Stunning the heart triggers changes in cardiac myocytes and coronary perfusion [6]. Status epilepticus activates the autonomic nervous system, increasing sympathetic nervous system control of cardiac function during seizure activity [7]. That is how it is believed that SE leads to SCM.

The diagnosis of SCM is based on seven diagnostic criteria that were put by the European Society of Cardiology [8]. Our patient met six of those criteria (transient regional wall motion abnormality, physical stressful trigger, extended regional wall motion abnormality beyond a single epicardial vascular distribution, the absence of culprit atherosclerotic coronary artery disease, positive but relatively small elevation in cardiac activity, and recovery of ventricular systolic function on cardiac imaging at follow-up). Cardiac catheterization could not be done in our patient due to risks linked to her resistant status epilepticus; in addition, other diagnostic information points to SCM as a likely diagnosis. However, cardiac catheterization is not mandatory in typical SCM [9]. Resolution of cardiac dysfunction ultimately occurred within five days, excluding the need for cardiac catheterization. The decision not to catheterize the coronary arteries was made on clinical risk-benefit analysis of patient safety. Vasospasm and plaque rupture could not be excluded in this case, due to the resolution of cardiac complications prior to establishing a seizure-free baseline. However, our patient met all the other criteria for SCM, and resolution of cardiac injury occurred within five days, making an infarct unlikely. This patient never required hemodynamic pressor augmentation, making cardiogenic shock very unlikely.

To our knowledge, only a few case reports were found on PubMed and Google Scholar describing SCM secondary to SE (Table 1). We used the terms ‘Takotsubo cardiomyopathy,’ ‘stress cardiomyopathy,’ and ‘broken heart syndrome,’ with ‘status epilepticus.’ We believe that this is the first case to describe SCM triggered by status epilepticus secondary to Herpes simplex virus encephalitis.

**Table 1:** Cases of stress cardiomyopathy secondary to status epilepticus * Cases that were diagnosed with stress cardiomyopathy without going to the cath lab

Name of author	Age/sex	Type of status epilepticus	Reason for SE	Days from the onset of SE to the echocardiogram	Echocardiographic findings	Time to normalization
Miller et al [9]*	49/f	Convulsive	Cerebral hypoxia or metabolic abnormalities in the setting of surgery	On the same day	Posterior wall akinesia, septal wall dyskinesia, and an ejection fraction (EF) of 15–20%	4 days
Shimizu et al[10]	82/f	Convulsive	Chronic grand mal epilepsy, starting when she was 20 years old.	1	Abnormal left ventricular wall motion with apical ballooning and basal hyperkinesis	4 weeks
Seow et al [11]	62/M	Convulsive	Encephalomalacia of the right basal frontal lobe with old lacunas in bilateral external capsules, bilateral corona radiate, and right hemipons.	On the same day	mildly impaired left ventricular ejection fraction of 40% with mid-ventricular ballooning and relative sparing of the apex	6 weeks
Legriel et al [12]	54/F	Convulsive	Temporo-occipital stroke 2 years earlier	On the same day	Latero-septo-apical hypokinesia with apical ballooning and a left ventricular ejection fraction (LVEF) of 40%.	1 week
Bosca et al [13]	61/F	Convulsive	Mesial temporal sclerosis	On the same day	Lateral–apical hypokinesis	12 days
Fugate et al [14]	82/F	Convulsive	Posterior reversible encephalopathy syndrome	On the same day	Apical akinesis	2 weeks
Traulle et al [15]	50/F	Convulsive	frontal syndrome with right facial palsy following a traumatic brain injury	2 days	Apical akinesis	Few days
Wakabayashi et al [16]*	68/F	Convulsive	Unable to determine	1 day	Mid to apical segmental akinesis	40
Benyounes et al [17]	79/F	Non-convulsive	Periventricular leukoencephalopathy	1 day	Apical hypokinesis	10 days
Finsterer et al [18]	47/F	convulsive	Encephalomyopathy	On the same day	Apical and mid-ventricular akinesis	28 days
Hocker et al [19]	18/F, 47/F, 25/M	Not able to determine	Not able to determine	4, 12, 11	EF 40% Global hypokinesis, Mild right ventricle dysfunction, EF 48% Generalized left ventricle dysfunction, EF 49% Mild generalized hypokinesis, Mild right ventricle dysfunction	55 days, 37 days, 22 days
Koo et al [20]	83/f	Convulsive	Cerebral hemorrhage on the left parietal and right temporal lobe	On the same day	Mid to apical left ventricular (LV) akinesia and right ventricular (RV) apical hypokinesia	10 days
Uemura et al [21]	61/f	Nonconvulsive	Unable to determine	Unable to determine	Apical akinesis	Unable to determine
Sakuragi et al [22]	59 YO F	Non-convulsive	Astrocytoma in her left temporal lobe	On the same day	Mild left ventricular dysfunction with severe hypokinesis or dyskinesis of the anterior and apical walls	30 days
Nandal et al [23]	71/F	Convulsive	ICH	Not done	Not done	Not done
Fawaz et al [24]	19/F	Convulsive	Synthetic cannabinoid known as ‘space’	3	EF of 16% and apical ballooning. The basal portions of the ventricular walls were hyperdynamic, while the mid to apical regions were akinetic	7 days

In this literature review, it was found that stress cardiomyopathy is more common after convulsive status epilepticus [9-24]. A STAT echocardiogram is very important in order to assess the myocardial injury and to have baseline imaging results. The time to resolution also remains unknown, as we do not know the exact left ventricular recovery mechanism, therefore close follow-up with repeat TTE is important. From our review, it was found that the time of normalization ranges from four days to about eight weeks [9-24]. The prevalence of SCM secondary to SE is much more common in women, especially postmenopausal women [9-24]. It is hypothesized that estrogen protects the heart from catecholamines.

SCM was initially believed to represent a benign pathology attributable to its self‐limiting clinical course. However, more data is coming up suggesting that the perception of SCM as having a benign prognosis should be generally ruled out. The risk of severe in-hospital complications is like that in patients with acute coronary syndrome. The complications, ranging from cardiogenic shock, the use of invasive or noninvasive ventilation, cardiopulmonary resuscitation, to death, were 19.1% in patients with SCM versus 19.3% in patients with acute coronary syndrome [25]. In-hospital mortality rates have ranged from 0% to 8% [25]. Our review demonstrated different causes for status epilepticus leading to SCM. Our case was the first case to make the connection between SCM and HSV encephalitis.

## Conclusions

This case demonstrates the importance of attentiveness and alertness in patients who develops acute cardiomyopathy in the setting of status epilepticus. Stress cardiomyopathy should be on the differential diagnosis in these instances. Serial echocardiographic evaluation is indicated and evaluation of possible ischemia is advised. Although further studies of the correlation between HSV encephalitis and SCM are warranted, we propose that including SCM in the differential diagnosis for patients with status epilepticus could lead to better management of patients.

## References

[1] Levitz RE (1998). Herpes simplex encephalitis: a review. Heart Lung.

[2] Zhang P, Yang Y, Zou J, Yang X, Liu Q, Yangmei Chen Y (2020). Seizures and epilepsy secondary to viral infection in the central nervous system. Acta Epileptologica.

[3] Tsuchihashi K, Ueshima K, Uchida T (2001). Transient left ventricular apical ballooning without coronary artery stenosis: a novel heart syndrome mimicking acute myocardial infarction. Japan. J Am Coll Cardiol.

[4] Y-Hassan S, Tornvall P (2018). Epidemiology, pathogenesis, and management of takotsubo syndrome. Clin Auton Res.

[5] Desai R, Singh S, Patel U (2020). Frequency of takotsubo cardiomyopathy in epilepsy-related hospitalizations among adults and its impact on in-hospital outcomes: a national standpoint. Int J Cardiol.

[6] Wittstein IS, Thiemann DR, Lima JA (2005). Neurohumoral features of myocardial stunning due to sudden emotional stress. N Engl J Med.

[7] Metcalf CS, Radwanski PB, Bealer SL (2009). Status epilepticus produces chronic alterations in cardiac sympathovagal balance. Epilepsia.

[8] Lyon AR, Bossone E, Schneider B (2016). Current state of knowledge on takotsubo syndrome: a position statement from the Taskforce on Takotsubo Syndrome of the Heart Failure Association of the European Society of Cardiology. Eur J Heart Fail.

[9] Miller GA, Ahmed YM, Tarant NS (2017). Recurrence of postoperative stress-induced cardiomyopathy resulting from status epilepticus. Case Rep Crit Care.

[10] Shimizu M, Kagawa A, Takano T, Masai H, Miwa Y (2008). Neurogenic stunned myocardium associated with status epileptics and postictal catecholamine surge. Intern Med.

[11] Seow SC, Lee YP, Teo SG, Hong EC, Lee CH (2008). Takotsubo cardiomyopathy associated with status epilepticus. Eur J Neurol.

[12] Legriel S, Bruneel F, Dalle L (2008). Recurrent takotsubo cardiomyopathy triggered by convulsive status epilepticus. Neurocrit Care.

[13] Bosca ME, Valero C, Pareja AI, Bonet M, Bosca I, Sanchez-Roy R, Ruvira J (2008). Tako-tsubo cardiomyopathy and status epilepticus: a case report. Eur J Neurol.

[14] Fugate JE, Wijdicks EF, Kumar G, Rabinstein AA (2009). One thing leads to another: GBS complicated by PRES and takotsubo cardiomyopathy. Neurocrit Care.

[15] Traullé S, Kubala M, Jarry G, Leborgne L, Hermida JS (2011). Tako-tsubo syndrome following status epilepticus. Neurol India.

[16] Wakabayashi K, Dohi T, Daida H (2011). Takotsubo cardiomyopathy associated with epilepsy complicated with giant thrombus. Int J Cardiol.

[17] Benyounes N, Obadia M, Devys JM, Thevenin A, Iglesias S (2011). Partial status epilepticus causing a transient left ventricular apical ballooning. Seizure.

[18] Finsterer J, Stöllberger C, Avanzini M, Bastovansky A, Keller H (2013). Aborted sudden unexplained death in epilepsy in a neuromuscular disorder leading to Takotsubo syndrome. Int J Cardiol.

[19] Hocker S, Prasad A, Rabinstein AA (2013). Cardiac injury in refractory status epilepticus. Epilepsia.

[20] Koo N, Yoon BW, Song Y, Lee CK, Lee TY, Hong JY (2015). Biventricular takotsubo cardiomyopathy associated with epilepsy. J Cardiovasc Ultrasound.

[21] Uemura J, Wada Y, Yagita Y (2016). Non-convulsive status epilepticus with takotsubo cardiomyopathy: a case report [Article in Japanese]. Rinsho Shinkeigaku.

[22] Sakuragi S, Tokunaga N, Okawa K, Kakishita M, Ohe T (2007). A case of takotsubo cardiomyopathy associated with epileptic seizure: reversible left ventricular wall motion abnormality and ST-segment elevation. Heart Vessels.

[23] Nandal S, Castles A, Asrar Ul Haq M, van Gaal W (2019). Takotsubo cardiomyopathy triggered by status epilepticus: case report and literature review. BMJ Case Rep.

[24] Al Fawaz S, Al Deeb M, Huffman JL, Al Kholaif NA, Garlich F, Chuang R (2019). A case of status epilepticus and transient stress cardiomyopathy associated with smoking the synthetic psychoactive cannabinoid, UR-144. Am J Case Rep.

[25] Uribarri A, Núñez-Gil IJ, Conty DA (2019). Short- and long-term prognosis of patients with takotsubo syndrome based on different triggers: importance of the physical nature. J Am Heart Assoc.

